# Patterns of Deterioration in Moderate Patients With COVID-19 From Jan 2020 to Mar 2020: A Multi-Center, Retrospective Cohort Study in China

**DOI:** 10.3389/fmed.2020.567296

**Published:** 2020-12-03

**Authors:** Sheng-long Chen, Hui-ying Feng, Hui Xu, Shan-shan Huang, Jiu-feng Sun, Lin Zhou, Jun-lei He, Wen-liang Song, Rui-jie Wang, Xin Li, Ming Fang

**Affiliations:** ^1^Guangdong Provincial People's Hospital, Guangdong Academy of Medical Sciences, Guangzhou, China; ^2^Center for Tuberculosis Control of Guangdong Province, Guangzhou, China; ^3^Shantou University Medical College, Shantou, China; ^4^Guangdong Provincial Center for Disease Control and Prevention, Guangdong Provincial Institute of Public Health, Guangzhou, China; ^5^The Second School of Clinical Medicine, Southern Medical University, Guangzhou, China; ^6^School of Medicine, South China University of Technology, Guangzhou, China; ^7^Guangdong Provincial People's Hospital-Nanhai Hospital, Foshan, China

**Keywords:** COVID-19, moderate, deterioration, time point, pattern

## Abstract

**Background:** Around the globe, moderate cases account for the largest proportion of all coronavirus disease 2019 (COVID-19) patients, and deteriorated moderate patients contribute the most in mortality. However, published articles failed to address the deterioration details of moderate cases, especially on when and how they deteriorated.

**Methods:** All moderate COVID-19 patients hospitalized in Guangdong Province from January 14 to March 16, 2020, were included in this multicenter retrospective cohort study and were divided into deteriorated and non-deteriorated groups according to clinical status. Symptoms and demographic, therapeutic, and laboratory test result characteristics were collected to explore the features of disease deterioration.

**Results:** Of 1,168 moderate patients included, 148 (13%) deteriorated to severe (130 cases) or critical (18 cases) status. Over 20% of the older subgroup (>50 years old) showed deterioration. The median time for deterioration was 11 days after onset [interquartile range (IQR) 9–14 days]. In addition, 12.2% severe cases could further develop to critical status after 3 days (IQR 2–6.5 days) of having a severe condition. Respiratory dysfunction and hypoxia were the major manifestations as disease deterioration, while 76 cases (52.1%) showed respiratory rate >30 breaths/min, 119 cases (80.4%) showed SaO_2_ <93%, 100 cases (67.5%) had 201 < PaO_2_/FiO_2_ < 300, and 27 cases (18.9%) had blood lactic acid >2.0 mmol/L. In view of multiple organ dysfunction, 87.8% of acute respiratory distress syndrome (ARDS), 20.2% of acute kidney injury (AKI), 6.8% of coagulopathy, 4% of acute heart failure (AHF), 3.4% of acute hepatic injury (AHI), and 5.4% of shock occurred in deteriorated patients, while organ injury occurred in the following sequence: ARDS, AKI, AHF, coagulopathy, AHI, and shock.

**Conclusions:** The deteriorated pattern of moderate COVID-19 patients is characterized as the 11th day from onset (IQR 9–14 days) being an important time point of disease deterioration with further exacerbation to critical condition in 3 days (IQR 2–6.5 days), A RDS followed by AKI being the typical modes of sequential organ damage.

## Introduction

Up to October 26, 2020, the rapid spread of coronavirus disease 2019 (COVID-19) has posed a great challenge to one's health and epidemic prevention in many countries and regions, and the number of infected people has exceeded 40 million (1). According to the existing reports and our practice, the largest proportion (84.25%) of diagnosed cases only had moderate symptoms at the beginning of admission, but their condition would worsen during hospitalization or even developed to critical status, which contributes most to mortality (2, 3). Therefore, avoiding death from disease deterioration in moderate patients is the most crucial strategy for COVID-19 treatment (4–6). There have been many researches on the deterioration of COVID-19 patients, but most of them were aimed at exploring the risk factors of deterioration (7–10). The population of those articles had not been clearly classified, and the clinical details for deterioration of COVID-19 have not been revealed either; hence, the guiding value of those studies for clinical treatment was very limited.

In view of the above, a multicenter observational study for the moderate population was carried out to describe the clinical features of moderate cases and determine time points of disease deterioration and the sequence of organ disorders explaining the following key issues: 1. What are the clinical characteristics of disease deterioration in moderate cases? 2. What is the time window and risk factors for disease deterioration in this population? 3. What is the order and degree of the main organ damage when patients deteriorate? These findings would provide specific clinical evidence for effective prevention and treatment of COVID-19.

## Methods

### Study Design

A multicenter retrospective cohort study was carried out at 32 hospitals in Guangdong Province designated to treat COVID-19 patients. All patients who were diagnosed with COVID-19 were screened for eligibility in our study. We retrospectively analyzed moderate patients from January 14, 2020, to March 16, 2020. The Guangdong COVID-19 Prevention and Control Headquarters was set up in Guangdong Province to direct and coordinate the treatment of COVID-19 patients across the province. An electronic medical information reporting system (E-System) was put together by the Guangdong Health Commission for the entire provincial medical data collection.

### Data Collection

Medical records for 1,315 COVID-19 patients in Guangdong province who had completed hospitalization by March 16, 2020, were extracted from the E-System. Medical records covered epidemiology, demographics, clinical symptoms, comorbidities, laboratory tests, and illness severity, as well as the main treatments during hospitalization and key dates in disease development. All patient data were reviewed by two researchers, and a third researcher adjudicated differences in interpretation if applicable.

### Definitions

Disease deterioration was defined as the development to severe or critical symptoms in moderate cases.

Moderate symptoms: fever and mild respiratory symptoms (cough, sore throat, runny nose, etc.), multiple patchy shadowing and ground-glass opacity in lung CT, and normal range of vital signs.

Severe symptoms: respiratory distress [respiratory rate (RR) ≥ 30 breaths/min and/or SaO_2_ ≤93% and/or arterial oxygen tension/inspiratory oxygen fraction (PaO_2_/FiO_2_) ≤300 mmHg under resting condition] (1 mmHg = 0.133 kPa) and/or radiology findings showing that the range of pulmonary lesions increased by more than 50% within 24–48 h, but no mechanical ventilation is required, and no organ failure.

Critical symptoms: Severe acute respiratory distress syndrome (ARDS) (PaO_2_/FiO_2_ ≤100 mmHg) and requiring mechanical ventilation and/or shock occurs and/or presence of organ failure.

Patients with moderate, severe, and critical symptoms were defined as moderate patients, severe patients, and critical patients, respectively.

Criteria of diagnosis and discharge: Patients were diagnosed with COVID-19 according to the Chinese management guidelines for COVID-19 (11). Laboratory procedures for high-throughput sequencing and real-time reverse transcriptase–polymerase chain reaction (RT-PCR) assays have been reported elsewhere (12, 13). Criteria for discharge were the absence of fever for at least 3 days, substantial improvement in lung CT, clinical remission of respiratory symptoms, and two negative severe acute respiratory syndrome coronavirus 2 (SARS-CoV-2) assays (throat swab samples) obtained at least 24 h apart.

ARDS was diagnosed according to the Berlin Definition (14). Acute renal injury [acute kidney injury (AKI)] was diagnosed according to the Kidney Disease Improving Global Outcomes (KDIGO) clinical practice guidelines (15). Coagulopathy was defined as a 3-s extension of prothrombin time or a 5-s extension of activated partial thromboplastin time. Acute heart failure (AHF) was diagnosed according to the European Society of Cardiology (ESC) (16). Acute hepatic injury (AHI) was diagnosed according to the European Association for the Study of the Liver (EASL) (17). Shock was defined according to the 2016. Third International Consensus Definition for Sepsis and Septic Shock (18). Multiple organ dysfunction syndrome (MODS) was defined as the simultaneous or sequential dysfunction of two or more organs in the course of acute diseases (19).

### Statistical Analysis

Continuous variables were presented as median with interquartile range (IQR) and were compared using Mann–Whitney U-test. Categorical variables were summarized as counts and percentages, and the two groups (showing deteriorated, showing non-deteriorated) were compared using chi-square tests or Fisher's exact tests. Missing data were not imputed. Univariate and multivariate logistic regression analyses were performed to explore pre-hospital risk factors associated with disease progression. The parameters of the logistic model are estimated using the maximum likelihood method. A two-sided α of < 0.05 was considered statistically significant. All analyses were performed using IBM SPSS software (version 25.0).

## Results

### Demographic and Basic Clinical Characteristics of the Study Population

**Figure 1:**
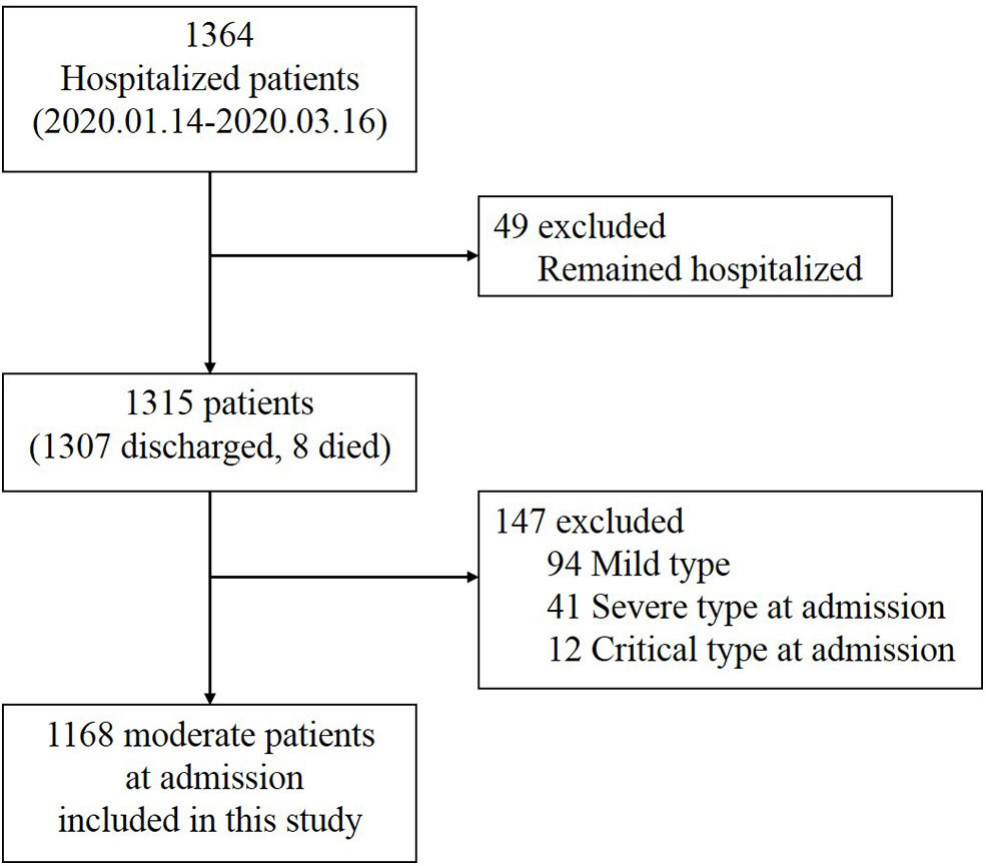
Selection of coronavirus disease 2019 (COVID-19) cases for inclusion in the study.

Of 1,364 patients diagnosed with COVID-19, 1,315 patients were enrolled in our study by excluding 49 patients who were still hospitalized. We further excluded 147 cases, including 94 mild cases that showed no signs of pneumonia on chest CT scans/X-rays throughout their hospital stay and 53 cases that presented with severe (41 cases) or critical (12 cases) symptoms at admission (Figure 1).

**Figure 2:**
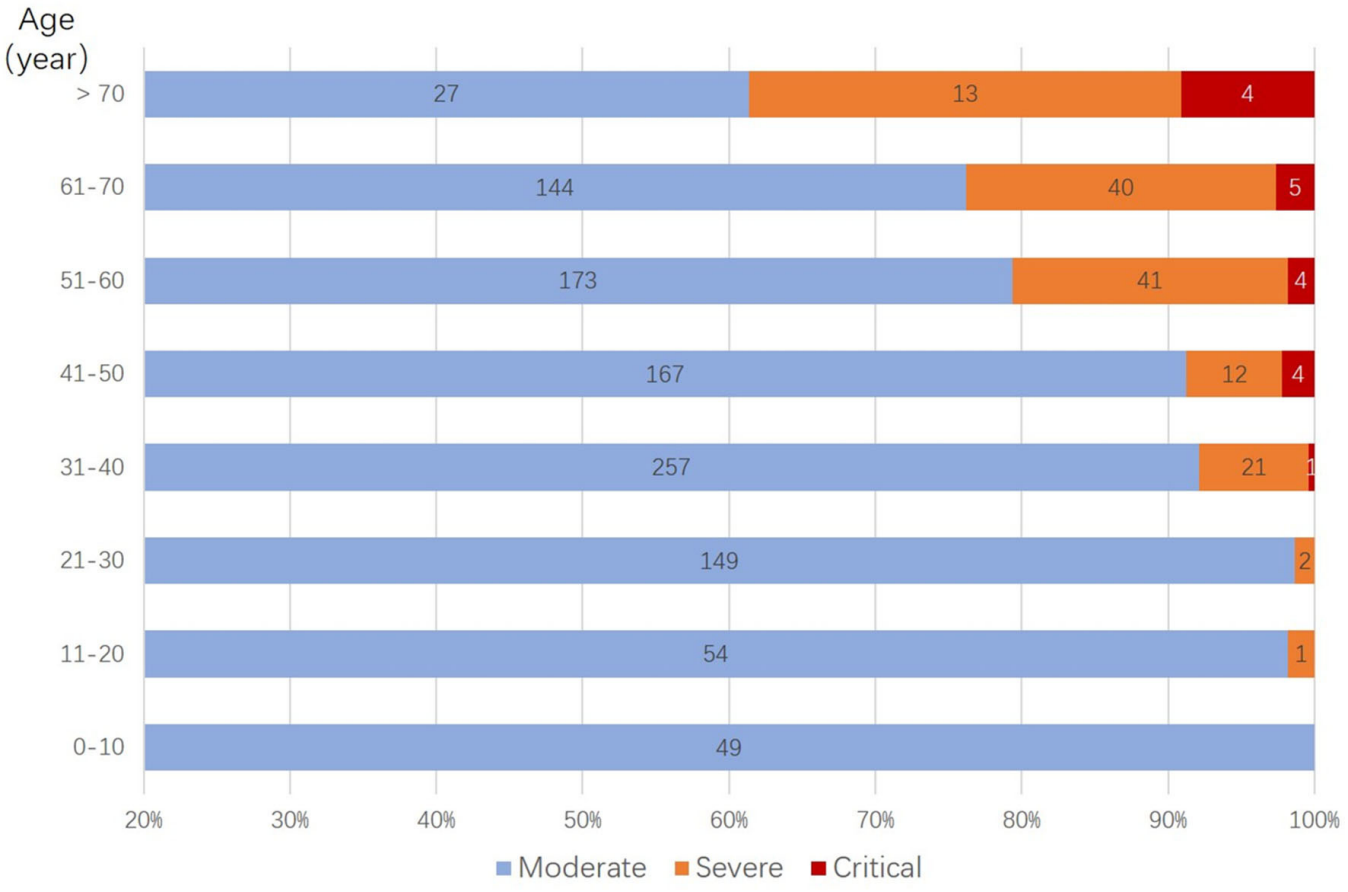
Distribution of deteriorated patients in different age groups. Stratified by age, more deterioration was observed with older age groups, in which cases older than 50 years have a significantly higher percentage of deterioration case (*p* < 0.001).

A total of 1,168 moderate cases at admission were included in the final analysis. Of these, 1,167 patients were successfully treated, and one patient died. Among them, 148 patients (13%) deteriorated during their hospital stay and developed severe (130 cases) or critical symptoms (18 cases). Compared with the non-deteriorated group, older median age [58.0 years (IQR 48.25–64) vs. 43.5 years (IQR 32–57), *p* < 0.001], higher proportion of male (46 vs. 61%, p = 0.001), higher proportion of comorbidities as diabetes (16 vs. 3%, *p* < 0.001), hypertension (24 vs. 7%, *p* < 0.001), cardiovascular disease (12 vs. 2%, *p* < 0.001), and cerebrovascular disease (3 vs. 1%, *p* = 0.028), and higher proportion of pre-admission symptoms as fever (89 vs. 66%, *p* < 0.001), cough (68 vs. 47%, *p* < 0.001), and dyspnea (16 vs. 5%, *p* < 0.001) were found in the deteriorated group (Table 1, Figure 2).

**Table 1:** Demographics and clinical characteristics of the 1,168 coronavirus disease 2019 (COVID-19) patients included in this study.

**Demographics and**	**Total**	**Non-deteriorated**	**Deteriorated (*****n*** **= 148)**			***p*-value**
**clinical characteristics**	**(*n* = 1,168)**	**(*n* = 1,020)**				
			**Severe patients**	**Critical patients**	**Total**	
			**(*n*_**1**_ = 130)**	**(*n*_**2**_ = 18)**		
Age, years	43.5 (32–57)	41 (31–56)	58 (48–64)	60.5 (48.25–69.5)	58 (48.25–64)	<0.001^*^
Age (≥50 years)	472 (40%)	363 (36%)	96 (74%)	13 (72%)	109 (74%)	<0.001^*^
Male	560 (48%)	470 (46%)	76 (56%)	14 (78%)	90 (61%)	0.001^*^
Imported case	915 (78%)	802 (79%)	98 (75%)	15 (83%)	113 (76%)	0.36
	(*n* = 1,155)	(*n* = 1,007)			(*n* = 148)	
**Comorbidity**						
Any	245 (21%)	172 (17%)	57 (44%)	16 (89%)	73 (49%)	<0.001^*^
Diabetes	50 (4%)	27 (3%)	19 (15%)	4 (22%)	23 (16%)	<0.001^*^
Hypertension	110 (9%)	74 (7%)	26 (20%)	10 (56%)	36 (24%)	<0.001^*^
Cardiovascular disease	33 (3%)	16 (2%)	14 (11%)	3 (17%)	17 (12%)	<0.001^*^
Cerebrovascular disease	10 (1%)	6 (1%)	3 (2%)	1 (6%)	4 (3%)	0.028^*^
Chronic kidney disease	11 (1%)	4 (0%)	5 (4%)	2 (11%)	7 (5%)	<0.001^*^
Chronic lung disease	33 (3%)	27 (3%)	6 (5%)	0	6 (4%)	0.30
Chronic liver disease	39 (3%)	31 (3%)	6 (5%)	2 (11%)	8 (5%)	0.14
History of Cancer	11 (1%)	8 (1%)	3 (2%)	0	3 (2%)	0.15
**Symptoms**						
Fever	807 (69%)	676 (66%)	114 (88%)	17 (94%)	131 (89%)	<0.001^*^
Cough	575 (49%)	475 (47%)	87 (67%)	13 (72%)	100 (68%)	<0.001^*^
Fatigue	132 (11%)	109 (11%)	19 (15%)	4 (22%)	23 (16%)	0.081
Myalgia	93 (8%)	80 (8%)	11 (9%)	2 (11%)	13 (9%)	0.69
Diarrhea	23 (2%)	20 (2%)	2 (2%)	1 (6%)	3 (2%)	>0.99
Dyspnea	78 (7%)	55 (5%)	17 (13%)	6 (33%)	23 (16%)	<0.001^*^
**Treatments during prodromal stage**						
Antibiotic	252	215 (21.1%)	32	5	37 (25.0%)	0.278
Antivirals	1,042	915 (89.7%)	115	12	127 (85.8%)	0.153
Oxygen therapy	1,106	959 (94.0%)	121	16	137 (92.6%)	0.492
Incubation, days	8 (5–13)	9 (5–13)	6 (3.25–9)	9 (5–12)	6 (4–10)	<0.001^*^
	(*n* = 748)	(*n* = 633)		(*n* = 115)		
Incubation (<8 days)	331 (28%)	261 (26%)	64 (49%)	6 (33%)	70 (47%)	<0.001^*^
	(*n* = 748)	(*n* = 633)		(*n* = 115)		
Disease onset to admission, days	3 (1–6)	3 (1–6)	4 (2–6.25)	3.5 (2.75–5)	4 (2–6)	0.004^*^
Length of hospitalization	18 (14–24)	17 (13–23)	23 (18–32.25)	38.5 (29.25–46)	24 (19–34)	<0.001^*^

*Data are median values [interquartile range (IQR)], n (%), or n/N (%). In comparison between deteriorated and non-deteriorated groups, p-values were calculated using the Mann–Whitney U-test, chi-square test, or Fisher's exact test, as appropriate*.

* *A two-sided α of < 0.05 was considered statistically significant*.

### Pre-Hospital Risk Factors Associated With Coronavirus Disease 2019 Deterioration

**Figure 3:**
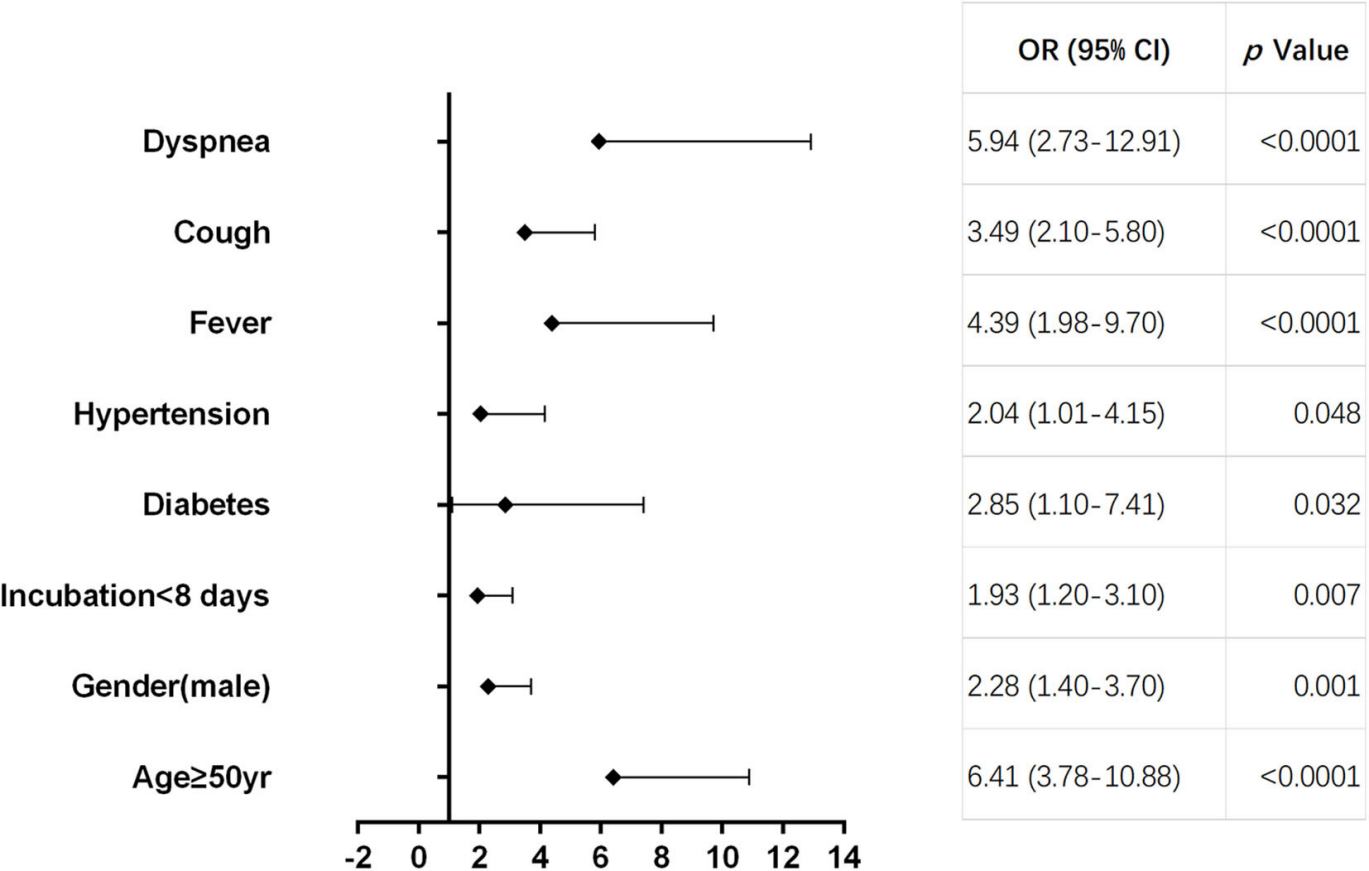
Forest plot of pre-hospital factors. Forest plot displaying the odds ratio (OR) and 95% confidence interval (95% CI) of each pre-hospital factor for deterioration in moderate cases.

As shown in Table 2, multivariate regression result suggested that age ≥50 years [odds ratio (OR) = 6.41, 95% CI 3.78–10.88, *p* < 0.0001], male (OR = 2.28, 95% CI 1.40–3.70, *p* = 0.01), incubation period <8 days (OR = 1.93, 95% CI 1.20–3.10, *p* = 0.007), diabetes (OR = 2.85, 95% CI 1.10–7.41, *p* = 0.032), hypertension (OR = 2.04, 95% CI 1.01–4.15, *p* = 0.048), fever (OR = 4.39, 95% CI 1.98–9.70, *p* < 0.0001), cough (OR = 3.49, 95% CI 2.10–5.80, *p* < 0.0001), and dyspnea (OR = 5.94, 95% CI 2.73–12.91, *p* < 0.0001) were pre-hospital risk factors of statistical significance based on filtrated items (*p* < 0.05) analyzed by univariate regression, while cardiovascular, cerebrovascular, and chronic kidney diseases were ruled out as pre-hospital risk factors. Forest plot displayed each pre-hospital factor for deterioration in moderate cases (Figure 3).

**Table 2:** Pre-hospital risk factors associated with deterioration in moderate coronavirus disease 2019 (COVID-19) cases.

**Variables**	**Univariate OR** **(95% CI)**	***P*-value**	**Multivariate** **OR (95% CI)**	***p*-value**
Age ≥50 years (vs. <50 years)	5.06 (3.43–7.45)	<0.0001^*^	6.41 (3.78–10.88)	<0.0001
Male (vs. female)	1.82 (1.28–2.58)	0.001^*^	2.28 (1.40–3.70)	0.001
Incubation <8 days (vs. ≥8 days)	2.22 (1.48–3.33)	<0.0001^*^	1.93 (1.20–3.10)	0.007
Disease onset to admission, days	1.05 (1.00–1.10)	0.032^*^	–	–
Diabetes	6.77 (3.77–12.16)	<0.0001^*^	2.85 (1.10–7.41)	0.032
Hypertension	4.11 (2.64–6.41)	<0.0001^*^	2.04 (1.01–4.15)	0.048
Cardiovascular disease	8.14 (4.02–16.51)	<0.0001^*^	–	–
Cerebrovascular disease	4.69 (1.31–16.84)	0.018^*^	–	–
Chronic kidney disease	12.61 (3.65–43.62)	<0.0001^*^	-	-
Fever	3.92 (2.33–6.61)	<0.0001^*^	4.39 (1.98–9.70)	<0.0001
Cough	2.39 (1.66–3.45)	<0.0001^*^	3.49 (2.10–5.80)	<0.0001
Dyspnea	3.23 (1.92–5.44)	<0.0001^*^	5.94 (2.73–12.91)	<0.0001

*OR, odds ratio*.

* *Variables significant at the 0.05 level in univariate analyses were considered*.

### Time Evolution for Deterioration in Moderate Patients

**Figure 4:**
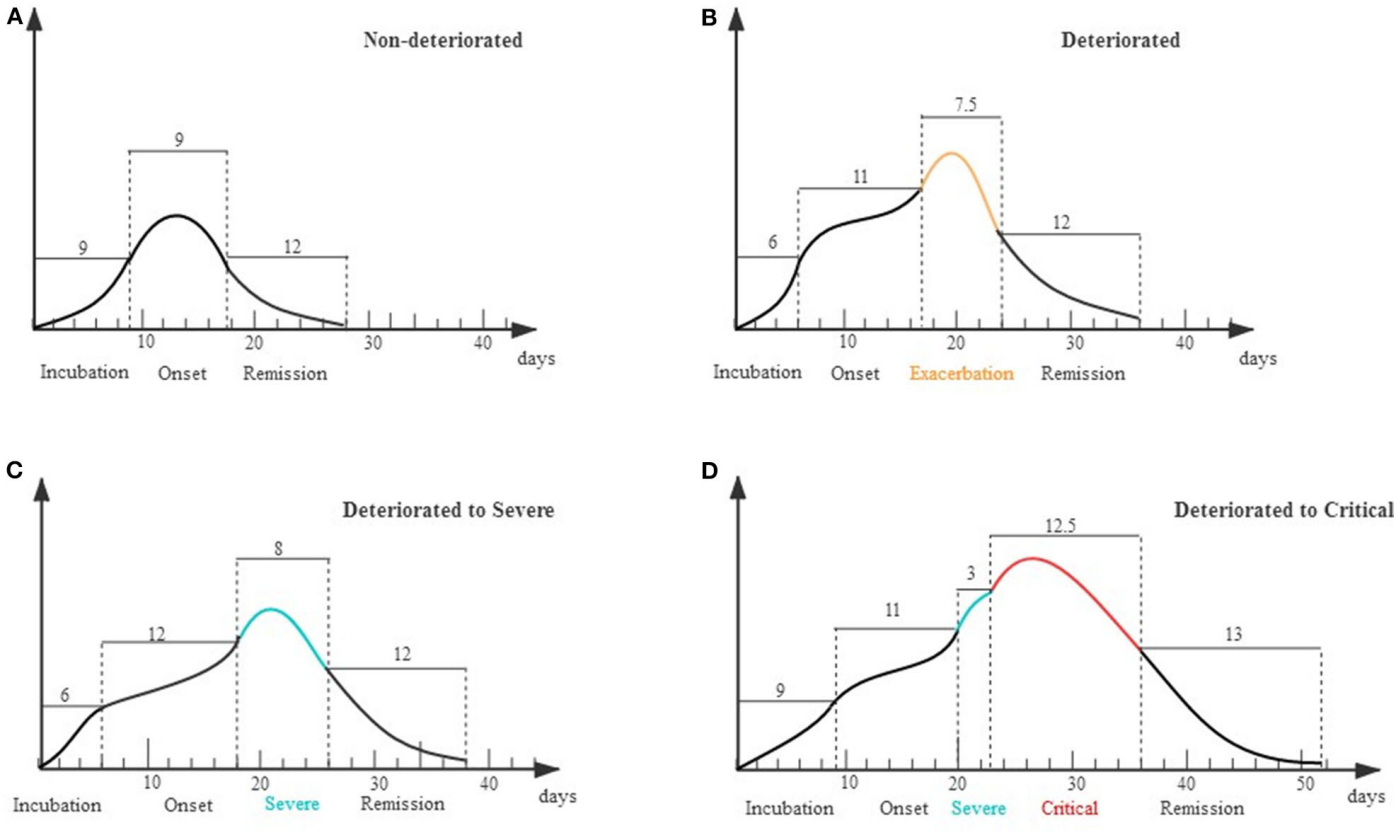
Schematic diagram for evolution of deterioration in moderate patients. X axis represents different stages of the clinical course that patients experienced; Y axis represents the severity of illness. **(A)** Non-deteriorated group. Patients typically had a 9-day disease incubation period [median, interquartile range (IQR) 5–13, *n* = 633] and a 9-day onset stage (IQR 7–12, *n* = 1,020) before entering a 12-day remission stage (IQR 9–17, *n* = 1,020). **(B)** Deteriorated group. Patients typically had a 6-day disease incubation period (IQR 4–10, *n* = 115) and an 11-day onset stage (IQR 9–14, *n* = 148) before deteriorating and entering an exacerbation stage of 7.5 days (IQR 4–12, *n* = 144). **(C)** Deteriorated to Severe. Patients had a 12-day onset stage (IQR 9–14, *n* = 130) before deteriorating to an 8-day exacerbation stage (IQR 4–12, *n* = 130). **(D)** Deteriorated to Critical. Patients deteriorated to a 3-day severe stage (IQR 2–6.5, *n* = 14) on the 11th day (IQR 8–12) from illness onset and further stepped into critical stage (IQR 6.75–25.75, *n* = 18).

The clinical course of each stage was displayed in a schematic diagram as Figure 4. For the non-deteriorated group, patients experienced 9 days (IQR 7–12) of onset stage and entered 9 days (IQR 9–14) remission stage. For the deteriorated group, patients worsen on the 11th day (IQR 9–14) after onset, and the deterioration period lasted for 7.5 days (IQR 4–12) (Figures 4A,B). In subgroup analysis, 130 cases only deteriorated to severe condition, of which deterioration happened on the 12th day (IQR 9–14) after onset. The other 18 cases deteriorated on the 11th day (IQR 8–12), while they suffered a second deterioration stage after 3 days (IQR 2–6.5) in severe condition (Figures 4C,D).

### Evaluation of Organ Injury Severity in Deteriorated Coronavirus Disease 2019 Patients

**Figure 5:**
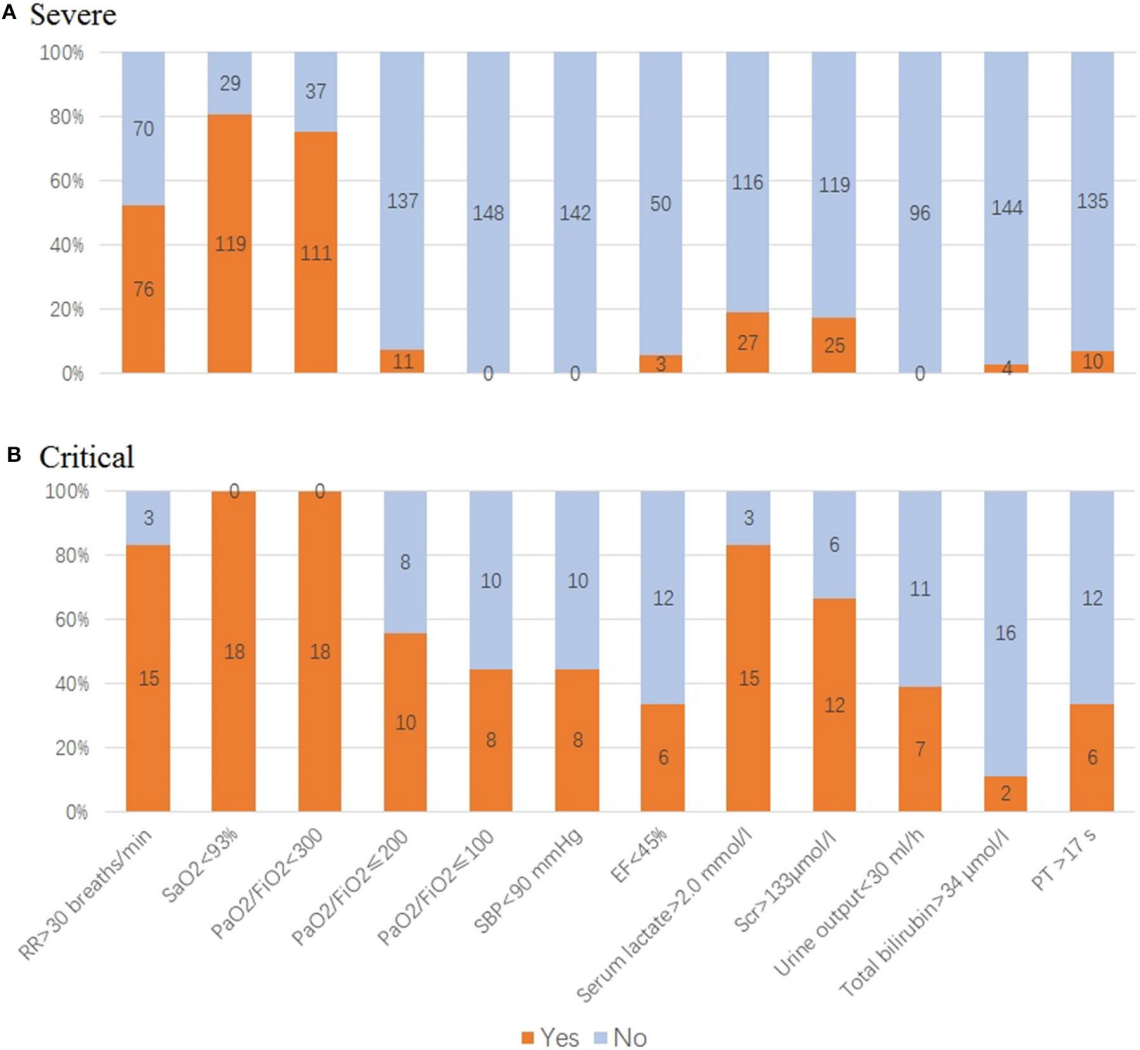
Evaluation of organ function in deteriorated coronavirus disease 2019 (COVID-19) patients. **(A)** For patients in severe condition: 76 cases with respiratory rate (RR) >30 breaths/min, 119 cases SaO_2_ <93%, 111 cases PaO_2_/FiO_2_ <300, 11 cases PaO_2_/FiO_2_ ≤200, 3 cases ejection fraction (EF) <45%, 27 cases serum lactate >2.0 mmol/l, 25 cases serum creatinine (Scr) >133 μmol/l, four cases total bilirubin >34 μmol/l, and 10 cases prothrombin time (PT) <17 s. **(B)** For patients in critical condition: 18 cases SaO_2_ <93%, PaO_2_/FiO_2_ <200, of which 44.4% cases PaO_2_/FiO_2_ ≤100, 83.3% cases serum lactate >2.0 mmol/L. Of those, 66.7% cases Scr >133 μmol/l, 38.9% cases urine output <30 ml/h. Acute circulatory failure and multiple organ dysfunction happened, with 44.4% cases having systolic blood pressure (SBP) <90 mmHg, 33.3% cases EF <45%, 11.1% cases total bilirubin >34 μmol/l, 33.3% cases PT <17 s.

Of 148 cases in the deteriorated group, 87.8% (*n* = 130) has progressed to severe condition while 12.2% (*n* = 18) to critical condition. When deterioration happened, respiratory dysfunction and hypoxia were major threats in our patients, with 76 cases (52.1%) having anRR >30 breaths/min, 119 cases (80.4%) SaO_2_ <93%, 100 cases (67.5%) 201 ≤ PaO_2_/FiO_2_ <300, 27 cases (18.9%) serum lactate (Lac) >2.0 mmol/L, and 25 cases (17.3%) serum creatinine (Scr) >133 μmol/l (Table 3, Figure 5A).

**Table 3:** Clinical features of 148 deteriorated moderate cases.

**Clinical variables**	**Severe**	**Critical**
RR (breaths/min)	33 (25–35) (*n* = 143)	33 (35–43) (*n* = 18)
>30, n (%)	76 (52.1)	15 (83.3)
SaO_2_ (%)	92 (90–92) (*n* = 148)	88 (85–91) (*n* = 18)
<93, n (%)	119 (80.4)	18 (100.0)
PaO_2_/FiO_2_ (mmHg)	230 (213–267) (*n* = 148)	109 (89.7–123) (*n* = 18)
>300, n (%)	37 (25.1)	0
201–300, n (%)	100 (67.5)	0
101–200, n (%)	11 (7.4)	10 (55.6)
≤100, n (%)	0	8 (44.4)
SBP (mmHg)	121 (112–145.5) (*n* = 142)	114 (87.7–133.7) (*n* = 18)
<90, n (%)	0	8 (44.4)
EF (%)	65 (56–67) (*n* = 53)	55.5 (41–62.5) (*n* = 18)
<45, n (%)	3 (5.6)	6 (33.3)
Serum lactate (mmol/l)	1.7 (1.4–1.9) (*n* = 143)	3.3 (2.3–4.4) (*n* = 18)
>2.0, n (%)	27 (18.9)	15 (83.3)
Scr (μmol/l)	98 (88–123) (*n* = 144)	209.5 (128.7–323) (*n* = 18)
>133, n (%)	25 (17.3)	12 (66.7)
Urine output (ml/h)	90 (78–100) *n* = 63	45 (23.6–58.2) (*n* = 18)
<30, n (%)	0	7 (38.9)
Total bilirubin (μmol/l)	21.1 (19.2–24.6) *n* = 148	24.2 (19.7–32) (*n* = 18)
>34, n (%)	4 (2.7)	2 (11.1)
PT (s)	15 (14–16) (*n* = 145)	14 (13–19) (*n* = 18)
>17, n (%)	10 (6.9)	6 (33.3)

*Data are median values (IQR), n (%), or n/N (%)*.

*RR, respiratory rate; SBP, systolic blood pressure; SaO_2_, blood oxygen saturation; PaO_2_/FiO_2_, arterial oxygen tension/inspiratory oxygen fraction; Scr, serum creatinine; EF, ejection fraction; PT, prothrombin time*.

In patients in critical condition, their lung function and hypoxia were even worse, with 18 cases having SaO_2_ <93%, PaO_2_/FiO_2_ <200, of which 44.4% cases had PaO_2_/FiO_2_ ≤100, 83.3% cases Lac >2.0 mmol/L. Meanwhile, AKI were tensed, with 66.7% cases Scr >133 μmol/l, 38.9% cases urine output <30 ml/h. Acute circulatory failure and multiple organ dysfunction happened, with 44.4% cases having systolic blood pressure (SBP) <90 mmHg, 33.3% cases ejection fraction (EF) <45%, 11.1% cases total bilirubin >34 μmol/l, 33.3% (*n* = 6) prothrombin time (PT) <17 s (Table 3, Figure 5B).

### Sequential Organ Injury in Deteriorated Coronavirus Disease 2019 Patients

**Figure 6:**
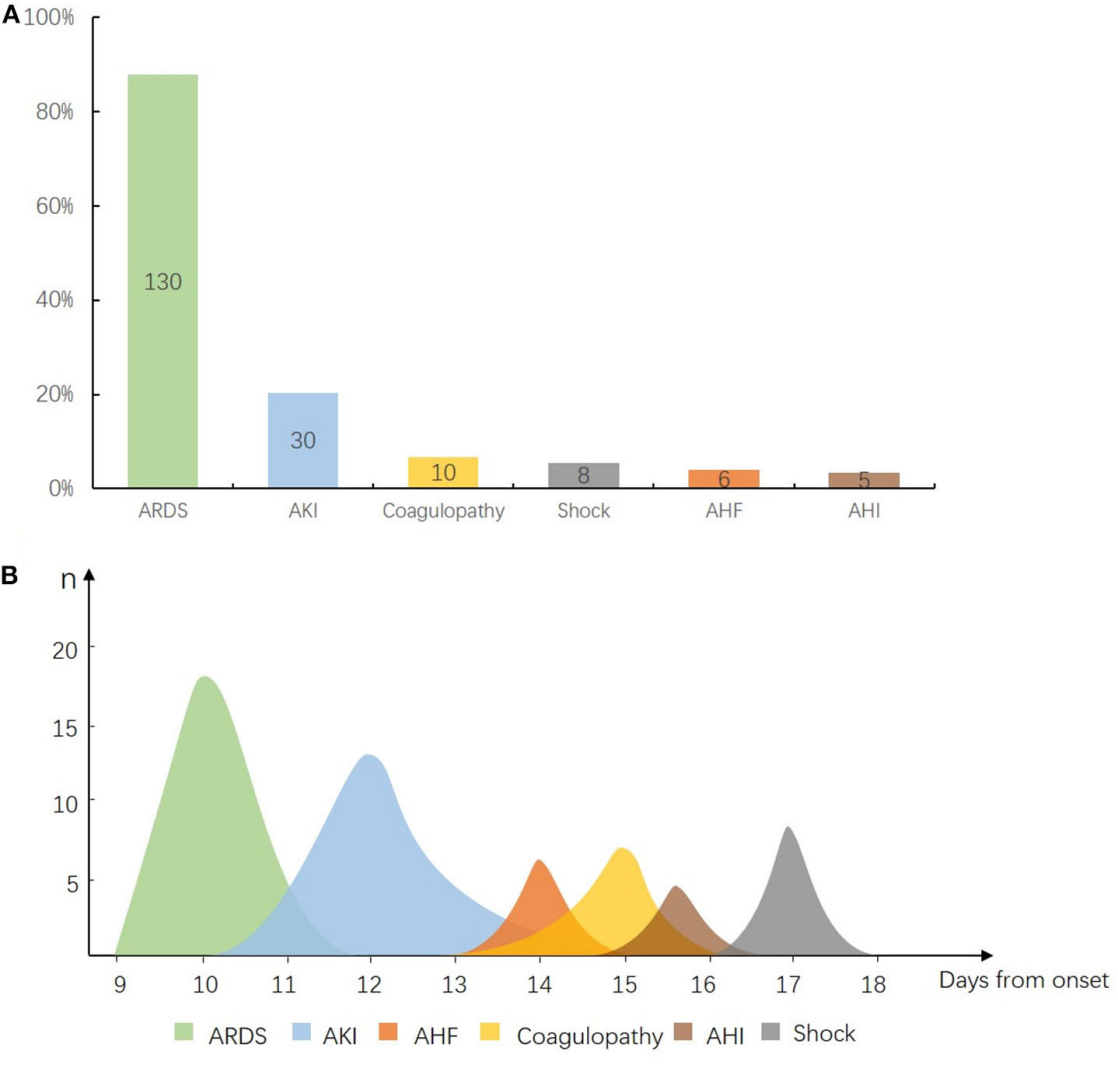
Sequential organ injury in deteriorated coronavirus disease 2019 (COVID-19) patients. **(A)** Composition of organ dysfunction in 148 deteriorated patients. X axis for each kind of organ injury and Y axis for the ratio of patients who developed each kind of organ injury. Out of 148 cases, 87.8% (*n* = 130) of acute respiratory distress syndrome (ARDS), 20.2% (*n* = 30) of acute kidney injury (AKI), 6.8% (*n* = 10) of coagulopathy, 4% (*n* = 6) of acute heart failure (AHF), 3.4% (*n* = 5) of acute hepatic injury (AHI), and 5.4% (*n* = 8) of shock. **(B)** Time windows for occurrence of organ injury in 18 critical patients. X axis for time from symptom onset to start of each kind of organ injury and Y axis for the number of patients who developed organ injury of each kind. ARDS occurred on day 10 [interquartile range (IQR) 9–12] from onset, AKI (*n* = 15) on day 12 (IQR 10–15), AHF (*n* = 6) on day 14 (IQR 13–15), coagulopathy (n = 7) on day 15 (IQR 13–16), AHI (*n* = 4) on day 15.5 (IQR 15–17), and shock (*n* = 8) on day 17 (IQR 16–18).

In view of multiple organ dysfunction (Figure 6A), 87.8% (*n* = 130) of ARDS, 20.2% (*n* = 30) of AKI, 6.8% (*n* = 10) of coagulopathy, 4% (*n* = 6) of AHF, 3.4% (*n* = 5) of AHI, and 5.4% (*n* = 8) of shock occurred in 148 deteriorated patients. Among them, the organ injuries in critical condition appeared on the following time sequence (Figure 6B): ARDS (*n* = 18) occurred on day 10 (IQR 9–12) from onset, AKI (*n* = 15) occurred on day 12 (IQR 10–15) from onset, AHF (*n* = 6) occurred on day 14 (IQR 13–15) from onset, coagulopathy (*n* = 7) occurred on day 15 (IQR 13–16) from onset, AHI (*n* = 4) occurred on day 15.5 (IQR 15–17) after onset, and shock (*n* = 8) on day 17 (IQR 16–18) from onset.

## Discussion

It was shown in our data that moderate cases accounted for the most (88.8%; 1,168/1,315) patients with COVID-19 in Guangdong Province. Moreover, the majority of severe and critical cases resulted from the deteriorated moderate cases (76.0%; 130/171 and 60.0%; 18/30, respectively). The results indicated that moderate cases were the main component of COVID-19 patients. Although several studies have elucidated the risk factors for deterioration in moderate patients, few information was provided on when and how disease deterioration happens (5–8).

Our data revealed that 148 patients, accounting for 13% of moderate cases, deteriorated to severe or critical condition. These patients had the marked characteristics of age >50 years old, male, and high proportion of combined chronic diseases, suggesting that these characteristics were related to disease deterioration. As reported by previous studies, these characteristics were the risk factors for mortality (20, 21). Thus, as these features were the inherent background of patients, they were worthy of clinicians' concern throughout the entire clinical process.

In our study, patients in the non-deteriorated group shifted into remission stage on 9 days (IQR 7–12) after symptom onset; however, patients in the deterioration group exacerbated on the 11th day (IQR 9–14) from onset. For the 18 patients who further exacerbated into critical status, only 3 days (IQR 2–6.5) were spent for exacerbation from severe condition. Data above were plotted in a schematic diagram to display. This suggested that the 2nd week from onset was the time window for deterioration, and it may further exacerbate to critical condition in 3 days since that. Study suggested that timely oxygen therapy or even humidified high-flow nasal cannula therapy be implemented to prevent deterioration (22). Hence, clinicians should pay more attention to the time points for deterioration—interfere in advance to prevent organ function disorders.

Regression results showed that age ≥50 years, dyspnea, fever, cough, diabetes, male gender, hypertension, and an incubation period <8 days were pre-hospital risk factors for deterioration in moderate COVID-19 cases. Several studies (20, 21, 23) had revealed that the prognosis was worse in older COVID-19 patients and those with more clinical symptoms like fever (?38.5°C), cough, and shortness of breath. Furthermore, they found that these COVID-19 patients had lower levels of lymphocyte and serum albumin, higher levels of lactate dehydrogenase, B-type natriuretic peptide, and D-dimer, which might be correlated with severity of illness. In addition, it is reported that a shorter incubation period for patients with acute respiratory syndrome coronavirus infection could be indicative of a higher infective dose, leading to faster/greater pathogen replication, outrunning adaptive immune responses, or leading to a more aggressive and damaging inflammatory response, therefore causing more severe disease (24). Hence, the above reasons could partly explain why the moderate COVID-19 patients with pre-hospital risk factors mentioned above were inclined to deteriorate. Although previous study had indicated that male gender might be prone to being affected (25), why disease deterioration occurred more in male COVID-19 patients is still unclear and more research is needed. Stated thus, it is important that clinicians should record patient symptoms and medical history in detail at the earliest opportunity. Patients with moderate COVID-19 symptoms and any of the above risk factors should be admitted to the hospital preferentially without delay and receive careful attention throughout the whole treatment process.

Then, what is the order and degree of the major organs involved during disease deterioration?

Pulmonary system was the primary target attacked by SARS-CoV-2. Our study showed that almost all deteriorated patients primarily appeared with shortness of breath, decreasing of SaO_2_ and oxygenation index, as well as increasing of serum lactate level, indicating that acute lung injury was the jumping-off point for disease deterioration in moderate COVID-19 patients. It has been reported that the viral spike glycoprotein of SARS-CoV-2 would specifically combine with angiotensin-converting enzyme-2 on the membrane of pulmonary epithelial cells when the virus invaded the lungs *via* the respiratory tract (26). Furthermore, histological examination after autopsy showed typical ARDS pathological manifestations in lung tissue of dead COVID-19 patients, such as bilateral diffuse alveolar damage accompanied by fibrous mucous exudates, exfoliation of pulmonary cells, and formation of hyaline membrane (27). In addition, a recent study found that the bronchoalveolar lavage fluid in severe/critical patients was enriched with macrophages derived from monocytes that produced multiple cytokines [interleukin (IL)-8, IL-6, and IL-1β] associated with inflammatory storm, and in the meantime, CD8^+^ T cells/proliferating T cell value apparently decreased (28). These immune disorders not only caused abnormal inflammatory response in the lung but also damaged the microcirculation of important organs besides the lung, even inducing disseminated intravascular coagulation (DIC) (29). These basic studies finely explained clinical phenomenon we observed and supported the treatment strategy of adequate oxygen.

Kidney was the second main target organ according to our data, as AKI accounted for 20.2% in deteriorated moderate cases and even 66.7% in critical patients. Through the scRNA-seq analysis, researchers found that podocytes and proximal tubules were potential host cells targeted by SARS-CoV-2, which caused AKI under the joint action of systemic inflammatory response induced by hypoxia (30, 31).

Furthermore, in this study, AHF, coagulation dysfunction, liver dysfunction, and circulatory failure occurred successively. These typical scenes of MODS in the intensive care unit (ICU) followed by further development of lung injury and tissue hypoxia (further reduced oxygenation index and increased lactate level), as well as renal injury, continuously worsened.

Therefore, from the overall clinical process, lung injury and AKI sequential development is an important pattern of COVID-19 progression, suggesting that clinicians should focus on hypoxia improvement and kidney protection at the early stage to prevent disease from developing MODS.

Our study has some limitations. First, by excluding patients who remained in the hospital on the cutoff date, we may have underestimated the effect of potential risk factors for predicting deterioration. Second, because a small number of patients cannot recall the date accurately of contacting with the transmission source, their exact incubation period is uncertain. Third, the lack of data in partial severe patients, like left heart EF, coagulatory function, urine volume, as well as deficiency of baseline organ function level in severe and critical patients prevents organ injury degree measuring, which is an inevitable flaw in a retrospective study. Further studies of cohort-based design will be more helpful for clinicians to understand the deterioration process.

In conclusion, our study revealed that COVID-19 patients with deterioration had the characteristics of older age, male, and more basic diseases. The deteriorated pattern of moderate COVID-19 patients was characterized as [1] the 11th day from onset (IQR 9–14) was an important time point of disease deterioration, and it may further exacerbate to critical condition in 3 days (IQR 2–6.5); [2] organ damage induced by COVID-19 with a certain sequence, and ARDS followed by AKI was the common mode of deterioration.

## Data Availability Statement

The data analyzed in this study was obtained from Guangdong Health Commission and is restricted to protect confidentiality. Requests to access these datasets should be directed to the corresponding author, and will only be provided with approval from the Guangdong Health Commission.

## Ethics Statement

The studies involving human participants were reviewed and approved by The Institutional Review Board of Guangdong Health Commission and Guangdong Provincial People's Hospital (No. GDREC2020028H). Written informed consent from the participants' legal guardian/next of kin was not required to participate in this study in accordance with the national legislation and the institutional requirements.

## Author Contributions

MF and XL had the idea for and designed the study. S-lC, HX, H-yF, and S-sH had full access to all data in the study and took responsibility for the integrity of the data and data analysis. S-sH, J-fS, J-lH, W-lS, and R-jW were responsible for data collection. MF, S-lC, H-yF, S-sH, J-fS, and LZ contributed to writing of the report. All authors contributed to data acquisition and data interpretation, reviewed, and approved the final version. All authors contributed to the article and approved the submitted version.

## Conflict of Interest

The authors declare that the research was conducted in the absence of any commercial or financial relationships that could be construed as a potential conflict of interest.
